# Applying enhanced recovery after surgery protocols in a patient with a giant spleen: a case report

**DOI:** 10.3389/fonc.2024.1422776

**Published:** 2024-08-15

**Authors:** Dan Fang, Biling Gan, Mei Li, Dailan Xiong

**Affiliations:** Department of Hepatobiliary Surgery, Department of General Surgery, Guangdong Provincial People’s Hospital (Guangdong Academy of Medical Sciences), Southern Medical University, Guangzhou, China

**Keywords:** enhanced recovery after surgery, splenomegaly, primary myelofibrosis, case report, nursing

## Abstract

Although splenomegaly is a common finding in several diseases, massive splenomegaly is rare. Patients with massive splenomegaly often present with a complex clinical picture. This case report describes a 72-year-old female with a complex medical history. Fifteen years ago, she was diagnosed with primary myelofibrosis, which subsequently led to progressive abdominal enlargement and bloating over the past 5 years. Recently, she developed edema in her limbs, accompanied by dizziness, shortness of breath, and fatigue. A massive splenomegaly was discovered during her hospitalization. Additionally, the patient has a history of Crohn’s disease, gout, renal insufficiency, and hypertension. Laboratory results reveal severe anemia and thrombocytopenia. Abdominal CT scans confirm the enlarged spleen and show ascites. She was treated by a multidisciplinary team comprising several departments. Even after a period of comprehensive treatment, the symptoms of massive splenomegaly did not significantly improve. Thus, the patient underwent an open surgical excision of the giant spleen. The weight of the giant spleen was 5.0 kg. During the perioperative period, Enhanced Recovery After Surgery (ERAS) protocols were applied to facilitate recovery. Her recovery was uneventful, and she was able to resume her regular daily routine shortly after the procedure. This report presented a complex and rare case of massive splenomegaly, and underscored that a proper medical and nursing care is the key to better recovery.

## Introduction

The spleen is the major site for blood production outside the bone marrow, particularly during periods of stress (1). Although splenomegaly is a common finding in several diseases, including liver diseases, hematological malignancies, and infection, massive splenomegaly is rare (2). One of the most common causes of massive splenomegaly is primary myelofibrosis (3, 4). Primary myelofibrosis is a chronic myeloproliferative neoplasm that causes extensive scarring in the bone marrow (5). Primary myelofibrosis leads to anemia, and as the disease advances, patients may develop pancytopenia (6, 7). Bone marrow fibrosis disrupts normal hematopoiesis impairing blood cell production (8). Splenomegaly causes increased blood cell sequestration and destruction. Alterations in iron metabolism further complicate anemia management. These factors collectively contribute to chronic anemia and even pancytopenia in primary myelofibrosis impacting patient quality of life and treatment strategies (9). Both splenomegaly and myelofibrosis can have significant impact on a patient’s health and quality of life (10). In addition to the array of burdens that patients with myelofibrosis may endure, they can also experience substantial disease-related discomfort (11). Moreover, patients with massive splenomegaly often present with a complex clinical picture. Given the complexity of these patients’ conditions and the potential for additional stress from surgical intervention, careful and effective nursing care is crucial.

Enhanced Recovery After Surgery (ERAS) protocols are multidisciplinary, evidence-based care pathways designed to reduce the patient’s surgical stress response, optimize their physiological function, and facilitate recovery (12). ERAS has the potential to improve patient outcomes, reduce healthcare costs, and enhance the overall quality of patient care. These protocols are increasingly being implemented across most surgical subspecialties due to their demonstrated effectiveness in promoting rapid and safe recovery postoperatively (13). Here, we present a rare case of a patient with massive splenomegaly secondary to primary myelofibrosis. The patient underwent a radical surgical resection, and ERAS was successfully implemented to facilitate recovery.

## Case report

A 72-year-old female was referred to our department due to “abdominal distension for over 5 years.” The patient was diagnosed with primary myelofibrosis 15 years ago. An abdominal CT scan 15 years ago revealed a thickened and enlarged spleen, although the exact measurements were not recorded. The patient mentioned undergoing additional abdominal CT scans or B ultrasound at local hospitals in recent years, with splenomegaly noted in her medical records. However, due to data-sharing restrictions, we were unable to access these images and specific data. Five years ago, the patient began to experience progressive abdominal enlargement and bloating, accompanied by dizziness and fatigue. Over the past 5 years, she has been maintained on 20 mg of ruxolitinib. She reported that the spleen size was slightly reduced, and symptoms were improved initially. However, over the last year, she experienced progressive fatigue and abdominal discomfort that limit her quality of life. Ruxolitinib was discontinued 1 month ago because of severe thrombocytopenia, prominent anemia, and failure to reduce spleen size. She also developed edema in the limbs, accompanied by dizziness, shortness of breath, and fatigue 1 month ago. She was admitted to the local hospital, and a massive splenomegaly was found. Then, the patient was referred to our department. The patient was diagnosed with Crohn’s disease 4 years ago. She has a history of gout, renal insufficiency, and hypertension for 3 years. She has been receiving long-term blood transfusion treatment.

**Figure 1:**
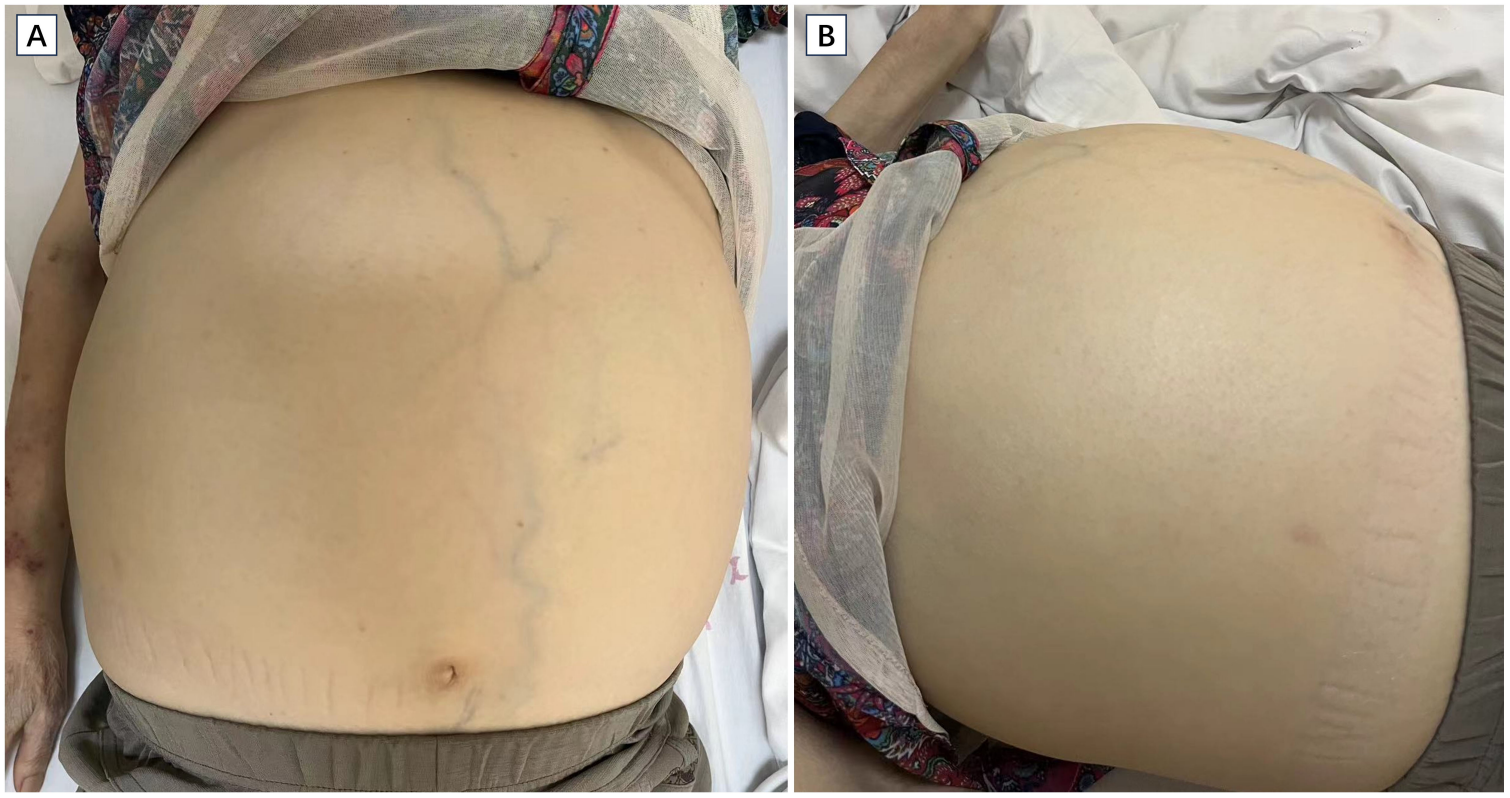
Images of the patient showing significant abdominal distension. The upper limb shows muscle wasting and patchy skin hemorrhage. Cross sectional **(A)** and coronal **(B)** view of CT scan revealing massive splenomegaly (asterisk).

On examination, her vital signs were stable, and her weight was 46 kg. The patient was thin, and muscle wasting was noticed, indicating cachexia. The abdomen was significantly distended (**Figure 1**), without tenderness or rebound tenderness. The liver was palpable 2 cm below the costal margin, and the spleen was palpated occupying mostly the left and lower abdominal quadrant. Stage 1 pressure ulcers were found on the buttocks. Scattered patchy skin hemorrhages were found on the upper extremities, and there was pitting edema on the lower extremities.

**Figure 2:**
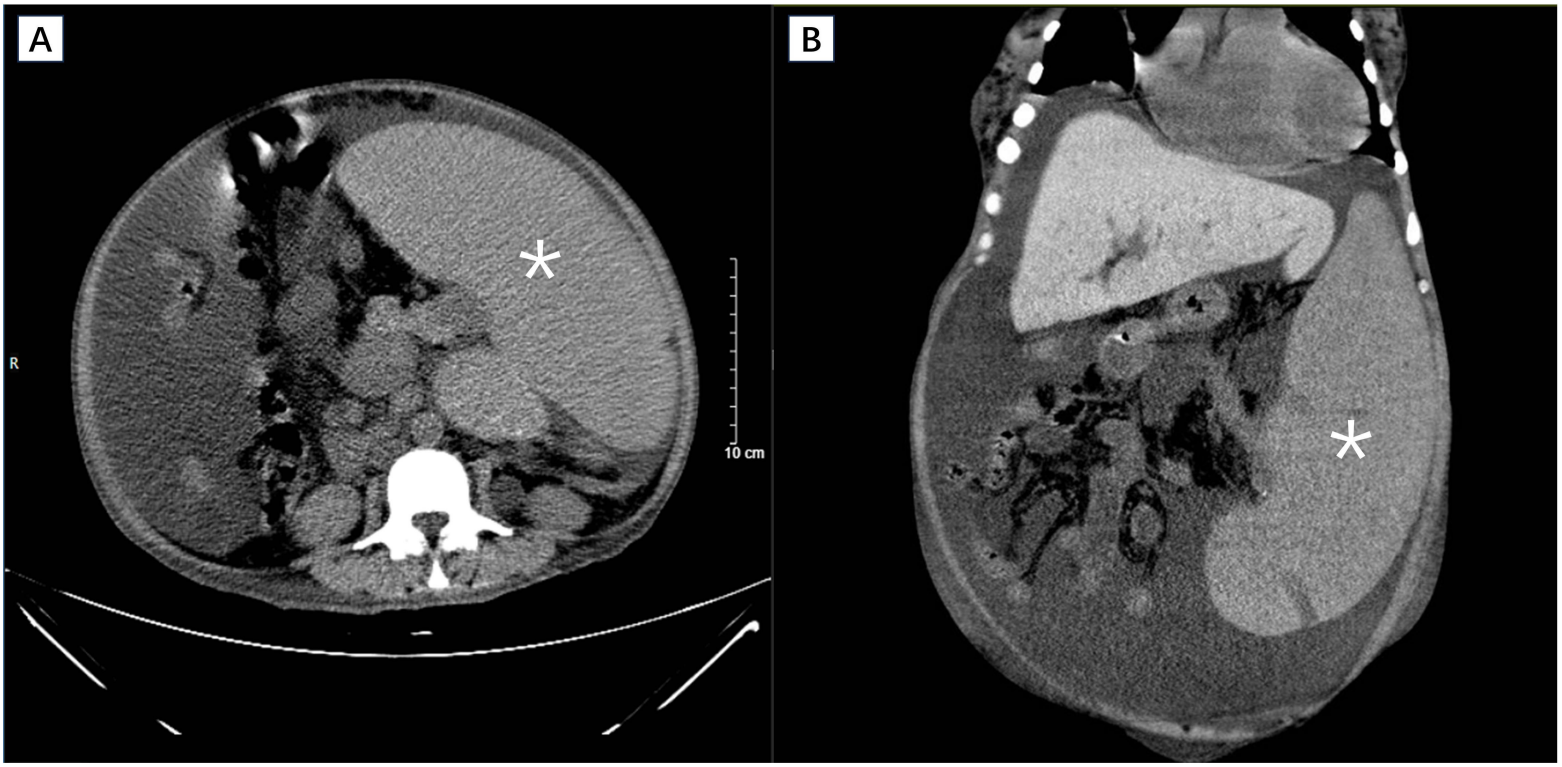
Cross sectional **(A)** and coronal **(B)** view of CT scan revealing massive splenomegaly (asterisk).

On admission, the auxiliary examination revealed severe anemia with a hemoglobin level of 49 g/L and thrombocytopenia with a platelet count of 49 × 10^9^/L. Additionally, the white blood cell count was 4.78 × 10^9^/L. Furthermore, the serum total protein was measured at 62.0 g/L, with an albumin level of 33.47 g/L (**Table 1**). Clotting parameters were recorded as follows: prothrombin time (PT) of 15.30 s, international normalized ratio (INR) of 1.18, prothrombin activity at 76.0%, activated partial thromboplastin time (aPTT) of 43.9 s, and plasma fibrinogen level of 2.57 g/L. The liver function test results are shown in **Table 1**. Abdominal CT scans revealed that the spleen was significantly enlarged (35 × 25 × 20 cm), the portal vein and splenic vein were widened, and large amounts of ascites were found in the abdominal and pelvic cavities (**Figure 2**). In addition, increased bone density in the lumbar vertebrae and pelvis were found showing ground-glass changes, consistent with myelofibrosis.

**Table 1:** Hematological laboratory findings.

Parameters	On admission	1 Day before splenectomy	1 Day after splenectomy	4 Days after splenectomy
White blood cell (×10^9^/L)	4.78	7.41	8.11	8.20
Hemoglobulin (g/L)	49	76	80	104
Platelet (×10^9^/L)	49	70	98	193
Total protein (g/L)	62.0	59.9	58.0	56.6
Albumin (g/L)	33.47	36.47	38.15	33.47
Total bilirubin (μmol/L)	29.9	23.6	18.0	17.9
Direct bilirubin (μmol/L)	10.8	9.5	8.4	5.7
Alanine aminotransferase (ALT) (U/L)	13	35	38	17
Aspartate aminotransferase (AST) (U/L)	11	50	44	21
Cholinesterase (U/L)	2754	3932	3173	2785

**Figure 3:**
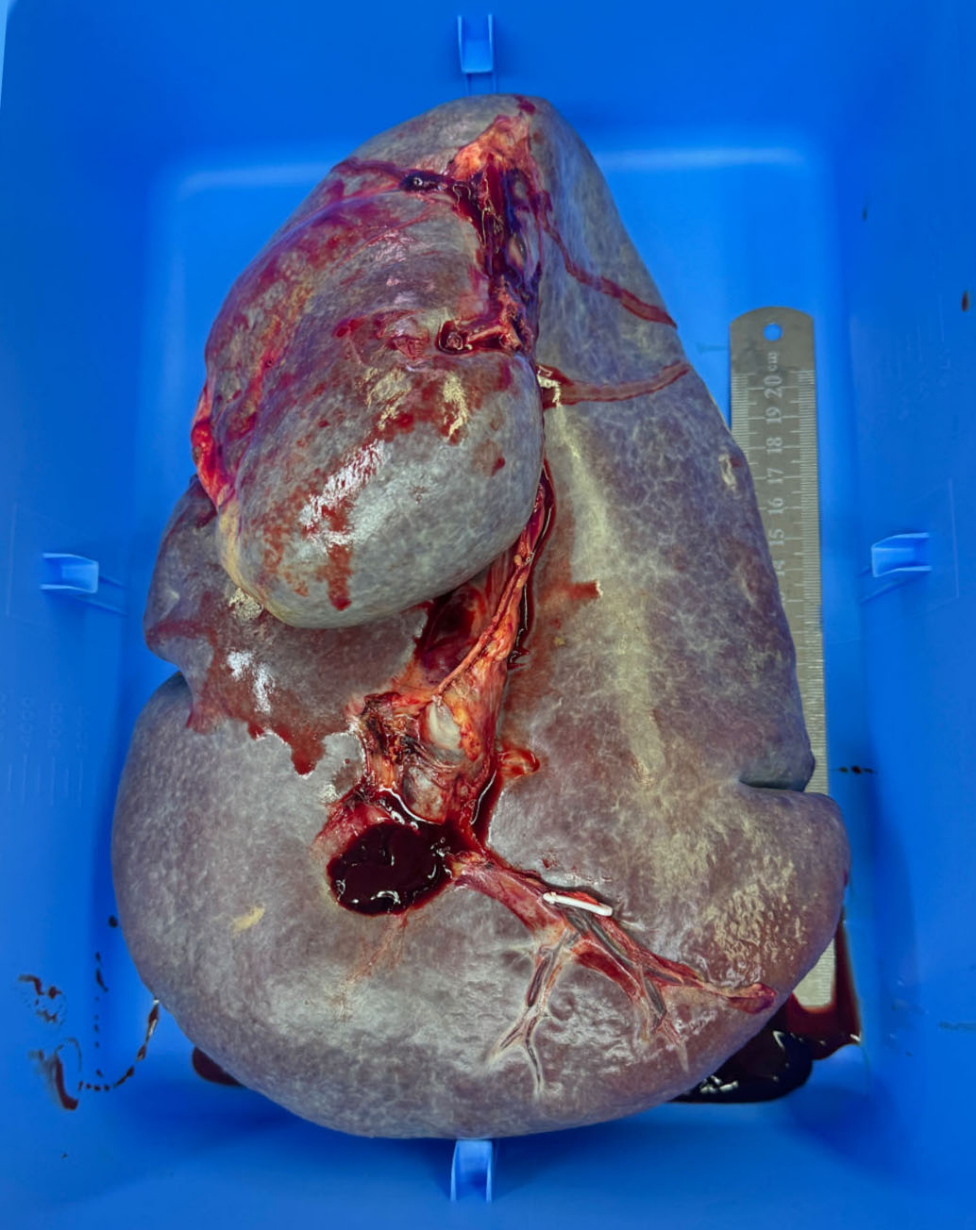
Histopathologic specimen of the giant spleen.

Upon diagnosis of massive splenomegaly, we organized a multidisciplinary team (MDT) comprising the departments of hematology, nephrology, cardiology, and anesthesiology for consultation. We formulated a comprehensive treatment plan that included medication and lifestyle adjustments. The patient underwent multiple blood transfusion to correct anemia and thrombocytopenia. The ascites was drained multiple times. After a period of comprehensive treatment, her hemoglobulin, platelet, and several liver function parameters improved (**Table 1**), but the symptoms of massive splenomegaly did not significantly improve. Given her history of ruxolitinib failure, prominent anemia, severe thrombocytopenia, and severe symptoms due to massive splenomegaly, splenectomy was the treatment of choice. Thus, the patient underwent an open surgical excision of the giant spleen (**Figure 3**). The weight of the giant spleen was 5.0 kg, and the postoperative weight of the patient was 34.0 kg. The patient was transferred to the Intensive Care Unit for postoperative resuscitation and remained there for the first 24 h. The patient was stable after the surgery and her hemoglobulin, platelet, and liver function parameters were significantly improved (**Table 1**). Her postoperative period was uneventful, and she was discharged 6 days after the surgery.

The patient suffered from severe anemia, malnutrition, thrombocytopenia, and stage 1 pressure ulcers, so nursing care was of great importance. During the perioperative period, ERAS protocols were applied to facilitate recovery. The ERAS protocol was implemented and included the following key components. 1) Patient/Family Education: At the day of admission, the patient and her family were educated about the diseases, procedure, expected recovery timeline, and the importance of active participation in the recovery process. 2) Patient Optimization: Guidance was provided focusing on nutrition, physical activity, and mental health. The patient was instructed to receive 30–35 kcal per kg per day and a protein intake of 1.5 g per kg per day through a standard nutrition regimen, with no requirement for protein restriction. Psychological support was provided to both the patient and her family. 3) Minimal Fasting and Preoperative Carbohydrate Loading: Preoperative fasting was reduced to 6 h for solids and 2 h for liquids. Carbohydrate loading was administered 2 h before the induction of anesthesia. Perioperative normothermia was maintained during the procedure. Traditionally, the patient was allowed to drink and eat 6 h after extubation. However, she was instructed to consume small amounts of water or semi-liquid food after extubation for 1 h to reduce postoperative hunger and thirst, and increase postoperative patient comfort. The patient did not develop any signs or symptoms of choking, postoperative aspiration, or gastrointestinal obstruction. We encouraged the patient to return to a normal diet as soon as possible. 4) Multimodal Analgesia and Early Mobilization: A multimodal analgesia approach was used to provide effective pain relief while minimizing opioid-related side effects. The Braden score of the patient was 11 indicating a high risk for developing pressure ulcer. Thus, following the surgery, the patient was encouraged to return to normal activities on the second day of surgery. This early mobilization was a key component to improve the pressure ulcer and ensure patient outcomes. The patient was glad to get rid of the burden caused by the giant spleen, and she was able to walk for a longer time. She was able to resume her regular daily routine shortly after the procedure, which underscores the efficacy of the ERAS protocol in this instance. Following her surgery, she was hospitalized for a duration of 6 days, during which she received additional treatments and engaged in rehabilitation. The rehabilitation continued even after her discharge from the hospital. Her weight increased to 50 kg 8 months after the surgery.

## Discussion

The definition of massive splenomegaly varies, and it is typically defined by most authors as a condition where the spleen crosses the midline, extends to the iliac crest, or weighs over 1.5 kg (3). The weight of the giant spleen in this case was 5.0 kg, which accounted for more than one-ninth of her body weight. The primary cause of splenomegaly had caused a significant burden on the patient. In addition to the array of burdens that patients with myelofibrosis may endure, they can also experience other substantial disease-related discomfort (11). They may suffer from severe anemia, malnutrition, tendency of hemorrhage and so on, all of which significantly impact their health and quality of life (14). Our patient also suffered from portal hypertension and liver failure. Increased hepatic blood flow or intrahepatic venous obstruction/stasis due to marked splenomegaly can lead to portal hypertension (15). Portal hypertension may cause liver damage due to increased hepatic resistance, sinusoidal damage, hepatic ischemia, and development of collateral circulation (16). In patients with primary myelofibrosis, the pathological study based on liver biopsy revealed hepatic myeloid metaplasia, an increased reticulin network, sinusoidal widening, and iron overload, despite the absence of prior blood transfusions (17). Iron overload may cause liver failure (18).

Splenectomy represents a potential therapeutic approach for addressing massive splenomegaly in myelofibrosis patients, particularly in cases resistant to conventional therapies. However, it carries significant risks including complications, morbidity, and mortality (19); thus, we generally select patients carefully prioritizing those with limited alternative treatment options. This procedure may be considered in patients experiencing substantial splenomegaly and related symptoms (20). The negative impact of splenomegaly on quality of life is so significant that reduction in spleen size is one of the top treatment goals for patients with myelofibrosis (21). However, the addition of surgical intervention can further exacerbate the physical and psychological stress experienced by these patients. Given the complexity of these patients’ conditions and the potential for additional stress from surgical intervention, careful and effective nursing care is crucial. The procedure benefited this patient greatly, but the decision to proceed with the surgery is complex and controversial due to the potential risks and benefits involved. To the best of our knowledge, there are currently no reported cases of ERAS protocol being applied to massive splenectomy. Studies have found that ERAS is effective in facilitating recovery for patients undergoing major abdominal surgery, and early extubation, early oral intake, mobilization, and multimodal-balanced analgesia are recommended (22–25).

The nutritional status of the patient was poor. It is considered as one of the independent factors that most influences the postoperative results in elective surgeries (26). The chronicity of her primary myelofibrosis, Crohn’s disease, and splenomegaly may contribute to it. The giant spleen may compress the nearby organs resulting in early satiety, which may further worsen the appetite of the patient. In addition to malnutrition, an extended perioperative fasting period has adverse effects on postoperative recovery (26). Thus, reducing the perioperative fasting time is important for this patient. It was found that the implementation of the ERAS protocol led to improved perioperative nutritional management and outcomes; early feeding was linked to reduced gastrointestinal symptoms without an increase in complications (27). Our patient resumed regular feeding shortly after surgery, and this strategy was successful.

Early mobilization is important for early recovery and avoids the progression of her stage 1 pressure ulcer. Weakness, muscle wasting, and the great burden caused by the giant spleen hinder the ability of mobilization of the patient and resulted in pressure ulcer. The giant spleen and ascites weighed more than 10.0 kg. Hypersplenism further contributed to anemia and thrombocytopenia. Severe thrombocytopenia may result in bleeding and exacerbate the anemia. Anemia contributed to both weakness and reduced mobilization. Studies reported that early mobilization based on the ERAS significantly reduces the risk of postoperative complications, accelerates functional walking capacity recovery, positively impacts various patient-reported outcomes, and shortens hospital length of stay (28). Therefore, recommending early mobilization according to ERAS protocols benefits both the early recovery and pressure ulcer of the patient.

In conclusion, massive splenomegaly is a rare complication of various diseases, and the clinical picture is complex. Comprehensive and careful management by a multidisciplinary team and nursing care according to ERAS are crucial to the patient’s recovery.

## Data Availability

The original contributions presented in the study are included in the article/supplementary material. Further inquiries can be directed to the corresponding author.
